# High turn-over rates at the upper range limit and elevational source-sink dynamics in a widespread songbird

**DOI:** 10.1038/s41598-021-98100-x

**Published:** 2021-09-16

**Authors:** Martin U. Grüebler, Johann von Hirschheydt, Fränzi Korner-Nievergelt

**Affiliations:** Swiss Ornithological Institute, Seerose 1, 6204 Sempach, Switzerland

**Keywords:** Behavioural ecology, Biogeography, Climate-change ecology, Population dynamics, Ecology, Ecology

## Abstract

The formation of an upper distributional range limit for species breeding along mountain slopes is often based on environmental gradients resulting in changing demographic rates towards high elevations. However, we still lack an empirical understanding of how the interplay of demographic parameters forms the upper range limit in highly mobile species. Here, we study apparent survival and within-study area dispersal over a 700 m elevational gradient in barn swallows (*Hirundo rustica*) by using 15 years of capture-mark-recapture data. Annual apparent survival of adult breeding birds decreased while breeding dispersal probability of adult females, but not males increased towards the upper range limit. Individuals at high elevations dispersed to farms situated at elevations lower than would be expected by random dispersal. These results suggest higher turn-over rates of breeding individuals at high elevations, an elevational increase in immigration and thus, within-population source-sink dynamics between low and high elevations. The formation of the upper range limit therefore is based on preference for low-elevation breeding sites and immigration to high elevations. Thus, shifts of the upper range limit are not only affected by changes in the quality of high-elevation habitats but also by factors affecting the number of immigrants produced at low elevations.

## Introduction

All species show limitations in their distribution and thus form distributional range limits^1,2^. Generally, the distributional range of a species is the consequence of spatial variation in demographic rates i.e. reproductive output, survival, emigration and immigration^3,4^. Variation in demographic rates in turn is based on spatial variation in biotic and abiotic factors^2,4–6^. Within this framework, theoretical studies showed that in situations of fixed environmental gradients, range limits may be additionally affected by differential dispersal patterns^3,7,8^.

Mountain slopes are typically characterized by strong climatic and environmental gradients over short distances^9^. Populations inhabiting high elevations are expected to evolve life-histories different from populations at low elevations as an adaptation to mountainous environments^1,10^. Recent reviews on life-history changes in relation to elevation revealed that high-elevation populations show consistently lower fecundity than low-elevation populations, but this productivity decrease is only balanced by higher adult survival rates in some cases^11,12^. A possible reason for this paradox might be that the increased fecundity in populations at low elevations is realized by a smaller fraction of the reproductively mature individuals due to intraspecific competition^12^. Alternatively, juvenile survival may be greater at high elevation compared to low elevation populations due to increased parental care or offspring body condition^13,14^.

Adaptations to mountainous environments may require sufficient genetic isolation from low elevation populations^15,16^. Alternatively, populations at the upper range limit can be sink populations maintained only by immigration from lower elevations thereby preventing adaptation^17,18^. Theoretical considerations^3,8,10^ and transplant experiments^18^ suggest that in species with high dispersal ability, range limits are shifted upwards beyond conditions supporting sustainable populations. Such species establish sink populations at the upper range limit producing source-sink dynamics over the elevational gradient^3,10–12^.

Upper range limits also often occur within populations, in particular in highly mobile species such as birds where populations cover large extents of elevations associated with environmental gradients^19^, connected by high rates of dispersal between high and low elevations. Knowledge of within-population elevational gradients of demographic rates rather than that of between-population differences in demography at varying elevations can help to understand the mechanisms underlying the formation of the upper limit of population distribution. It also contributes to the understanding of the demographic mechanisms resulting in the upward shifts of range limits observed in some mobile species due to climate change in mountainous areas^17,20–24^.

In birds, we still lack a comprehensive understanding of how distributional ranges are limited at increasing elevations and what is the role of spatial dynamics in the formation of the upper range limit. To understand these processes, researchers called for studies investigating several demographic rates across elevational gradients simultaneously in long-term studies^4,25,26^. Decreased fecundity or reproductive output at the upper range limit would suggest source-sink effects within populations^17,18^. However, adult mortality or emigration rates could also increase with elevation resulting in higher turn-over rates of individuals and increased dispersal movements away from the upper range limit which then only could be stabilized by increased settlement at the upper range limit.

Reduced reproductive output is not enough to form an upper range limit of a population if individuals disperse randomly. At least a preference for (better) breeding sites at lower elevations is additionally required. This might be the case in despotic settlement processes where individuals settling at high elevations are unable to occupy high quality, low elevation breeding sites at the moment of settlement^27,28^. In such situations, we expect downwards within-population dispersal (natal or breeding dispersal) and thus, either lower recruitment rates (natal dispersal) or higher turn-over rates of individuals (breeding dispersal) at high compared to low elevations. Here, we study the spatial variation in apparent survival and within-study area dispersal in an Alpine population of barn swallows (*Hirundo rustica*) in relation to the elevation of nest sites by using a long-term capture-mark-recapture data set. The study area includes potential breeding sites at elevations that exceed the current upper distributional range limit of the species by far. In a recent study, we showed that reproductive effort in terms of fecundity and multi-broodedness is greater, but that the annual reproductive output is reduced at high elevations^29^. While the decrease in annual reproductive output is assumed also for this study, increased reproductive effort might result in reduced survival due to reproductive costs. However, since reduced reproductive output has been shown to increase breeding dispersal rates in barn swallows^30^, we expect higher within-population dispersal rates downwards at high elevations. Our results contribute to the understanding of spatial processes in mountainous gradients restricting elevational distributions of birds.

## Results

### Recapture probability and apparent survival

**Figure 1:**
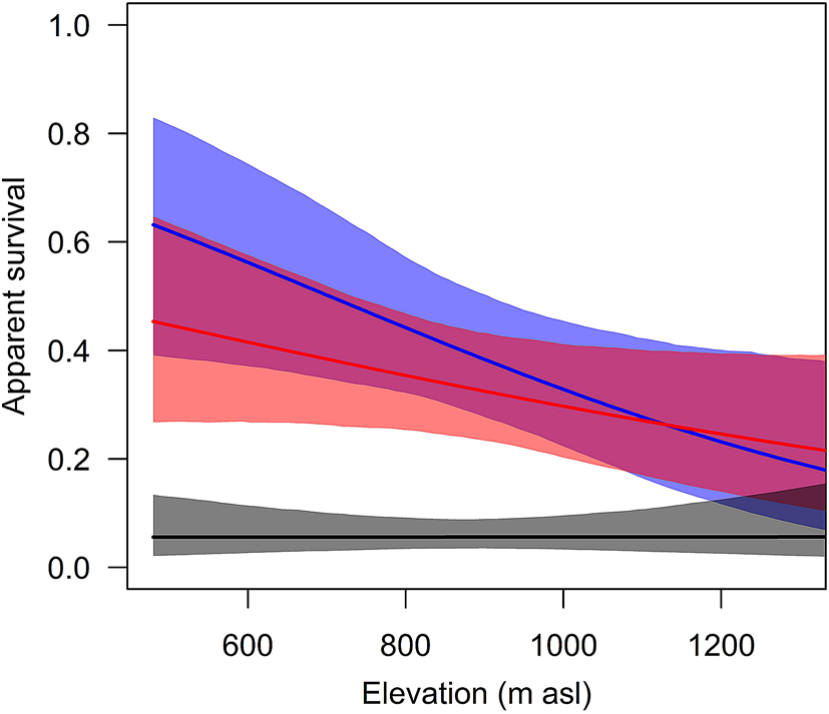
Model estimates of apparent survival probabilities in relation to elevation for females (red), males (blue), and juveniles (black). The shadowed areas indicate 95% credible intervals. N = 1531 ringed individuals (1337 nestlings, 194 adults) and 89 recaptured individuals.

During the study period, 1531 individuals (1337 nestlings and 194 adults) were ringed of which 89 individuals were recaptured in the study area at least once. Sex was known for adults and recaptured nestlings (n = 106 males and 129 females). For 1296 nestlings, sex was not known. Recapture probability was lower for first year birds than for older birds (Table 1). For older birds, recapture probability was essentially independent of elevation of the breeding site, whereas for first year birds, it decreased with increasing elevation. Apparent survival declined with elevation, with a clear effect in males but only a week effect in females (Table 1, Fig. 1). Apparent survival tended to be lower for females compared to males at low elevations and the stronger decrease in male apparent survival with elevation resulted in similar survival estimates between sexes at high elevations. Apparent survival of juveniles (i.e., recruitment) was low and independent of elevation (Table 1, Fig. 1).

**Table 1:** Parameter estimates (mean of the posterior distribution) of the survival model with 95% credible intervals (CrI).

Parameter	Estimate	95% CrI
**Recapture probability**		
Intercept	1.02	0.22 to 2.00
Elevation	− 0.02	− 0.67 to 0.72
Juvenile	− 1.83	− 2.98 to − 0.72
Elevation x Juvenile	− 0.28	− 1.35 to 0.76
Between-year SD	0.59	0.03 to 1.66
**Apparent survival probability**		
Intercept adult males	− 0.77	− 1.32 to − 0.22
Intercept adult females	− 0.89	− 1.42 to − 0.37
Intercept juveniles	− 2.82	− 3.02 to − 2.22
Elevation adult males	− 0.59	− 1.15 to − 0.08
Elevation adult females	− 0.32	− 0.72 to 0.08
Elevation juveniles	0.00	− 0.51 to 0.54
Location within study area	0.26	− 0.26 to 0.77
Between-year SD	0.21	0.01 to 0.63
Between-farm SD	0.20	0.01 to 0.55
**Mixture model for sex**		
Proportion of males	0.45	0.39 to 0.51

Estimates for recapture and apparent survival probability are on the log-odds scale. Estimated proportion of males among the non-identified individuals is on the proportion scale. N = 1531 ringed individuals (1337 nestlings, 194 adults) and 89 recaptured individuals.

### Dispersal probability

**Figure 2:**
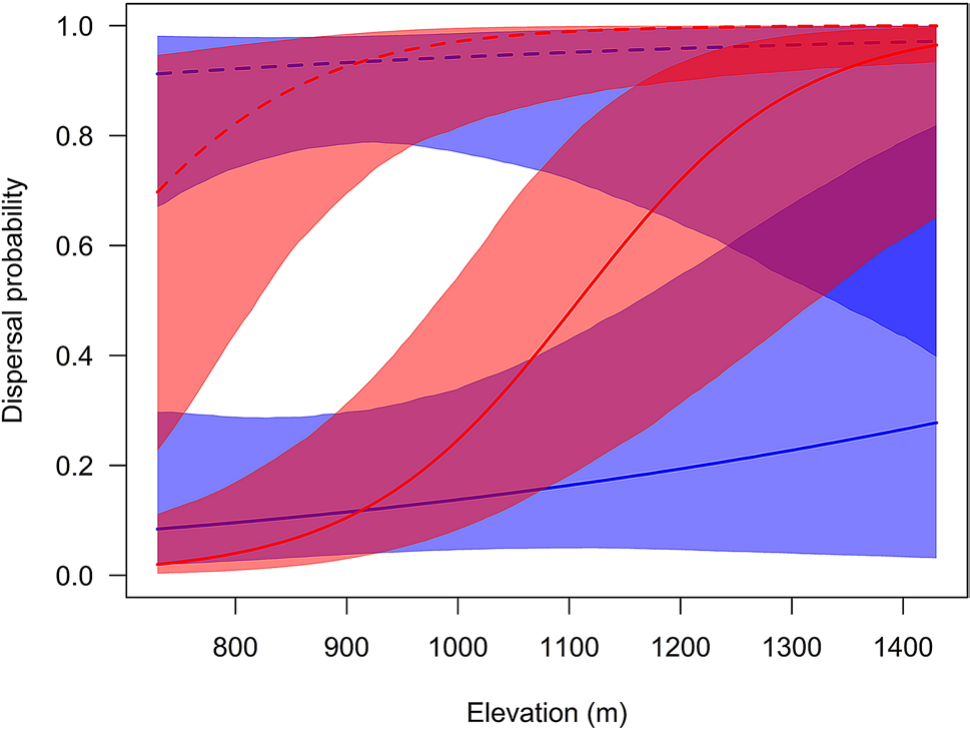
Model estimates of dispersal probabilities in relation to elevation for females (red), males (blue), adults (solid lines), and juveniles (broken lines). The shadowed areas indicate 95% credible intervals. N = 126 recaptures of 89 individuals.

Within-study area dispersal probabilities showed clear differences between age categories and were lower for adults compared to juveniles (Table 2, Fig. 2). Of recaptured juveniles, 91% dispersed from their natal farm (n = 43; 4 males returned to their natal farm), whereas only 17% of the adult breeding birds (n = 83) changed the breeding site from one to the next year. In adult females, dispersal probability clearly increased with increasing elevation, while the analysis showed no evidence for such a relationship in adult males and in juveniles (Table 2, Fig. 2). We found clear effects of the interactions of elevation with age and sex. This resulted in the following pattern: adult males and females did not differ in dispersal probabilities at low elevations, but dispersal probabilities of adult females increased with elevation and nearly reached the high dispersal rates of juveniles at the upper limit of the elevational range (Fig. 2).

**Table 2:** Parameter estimates of the binomial mixed model investigating factors affecting dispersal probability.

Parameter	Estimate	95% CrI
**Fixed effects**		
Intercept	1.88	0.39 to 3.35
Elevation (z-transformed)	0.36	− 1.04 to 1.74
Age (adult)	− 4.66	− 6.12 to − 3.16
Sex (female)	0.13	− 1.21 to 1.49
Location within study area	0.80	− 0.67 to 2.25
Elevation × sex (female)	1.75	0.38 to 3.04
Elevation × age (adult)	0.07	− 1.49 to − 1.68
**Random effects**		
Individual	< 0.01	< 0.01 to < 0.01
Location	< 0.01	< 0.01 to < 0.01
Year	0.34	0.21 to 0.48

The estimates are given as obtained from the Laplace approximation. The lower and upper limits of the 95% credible intervals are based on Monte Carlo simulation of the posterior distribution. For the random effects the among-group standard deviation is given. N = 126 recaptures of 89 individuals.

### Dispersal distances and corrected elevational shift of individuals

**Figure 3:**
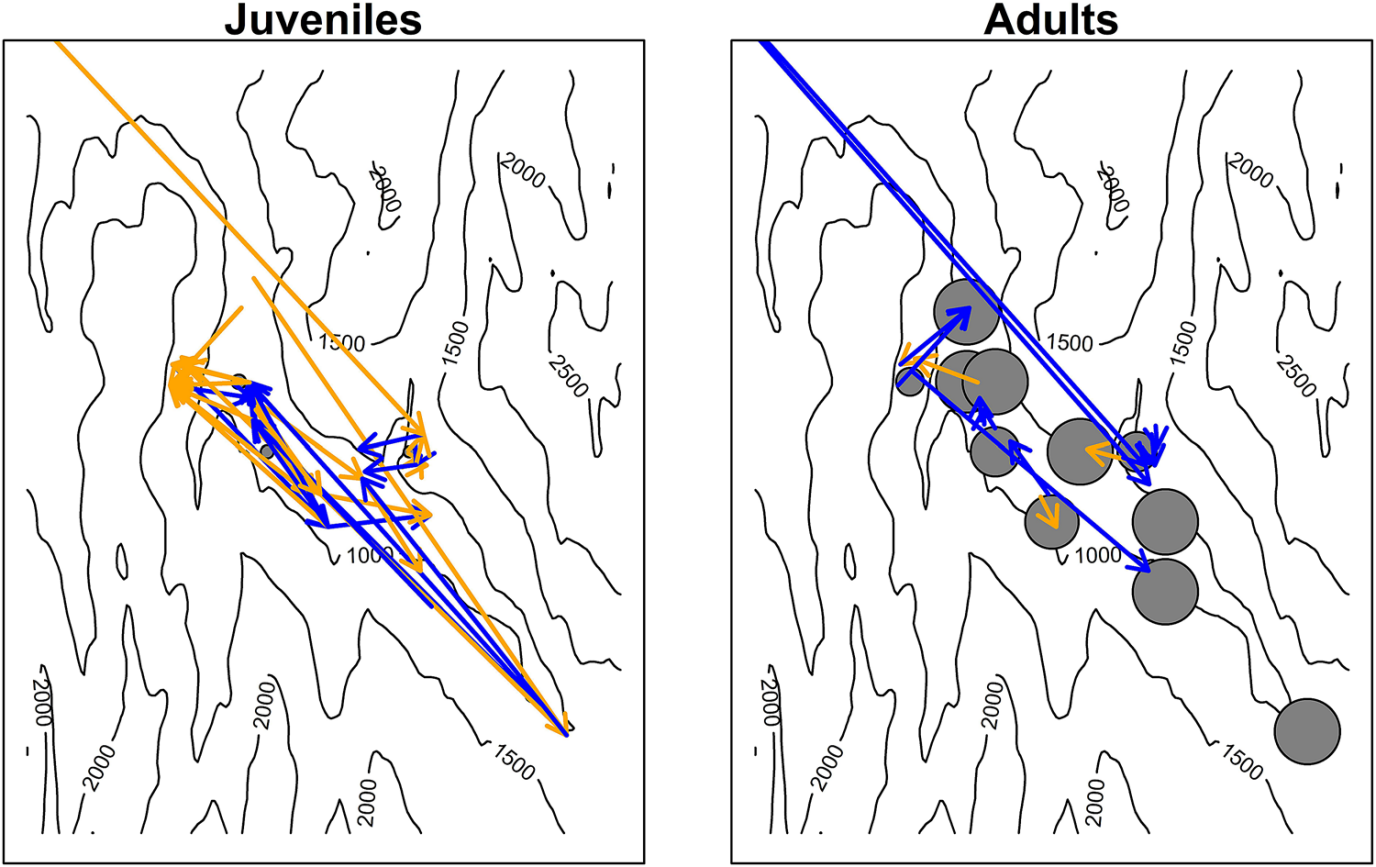
Study area. Sites with non-dispersers (grey circles, the larger the circle the larger the proportion of non-dispersers) and dispersal events (blue arrows: downwards dispersal; orange arrows: upwards dispersal) for juveniles (left panel) and adults (right panel) are shown. N = 126 recaptures of 89 individuals.

**Figure 4:**
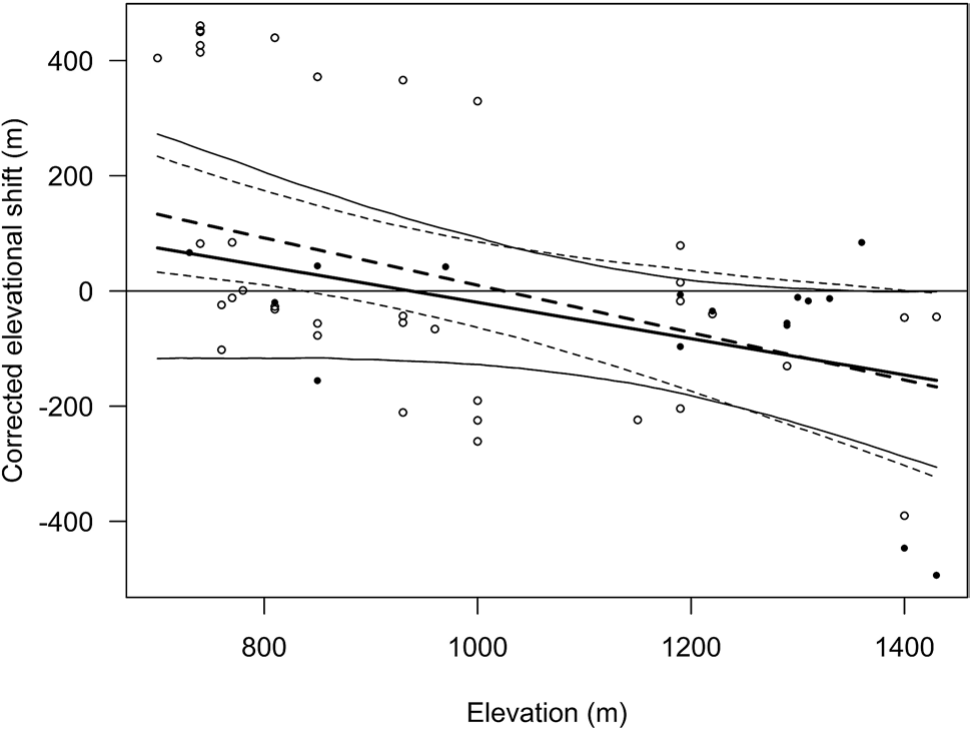
Corrected elevational shift (in meters) in relation to elevation for adults (solid dots and lines), and juveniles (open circles and broken lines). The 95% credible intervals are indicated by the outer lines. N = 56 dispersal events of 51 individuals from 26 farms.

The data set for the analyses of within-study area dispersal distances and corrected elevational shifts included 56 occasions of dispersal events with known start and end point (12 females, 5 males, 39 juveniles; 51 individuals from 26 farms). Breeding dispersal distances (adults) were smaller than natal dispersal distances (juveniles; breeding dispersal distance = 1.25 km, s.d. = 2.12 km, n = 17; natal dispersal distance = 4.12 km, s.d. = 3.56 km, n = 39; Table 3, Fig. 3). Credible intervals of estimated correlations between dispersal distance and elevation all included both medium to strong negative as well as medium to strong positive correlations. Consequently, we refrain from drawing conclusion from this case study. However, barn swallows breeding or fledged at the highest elevations dispersed to farms situated at lower elevations than the average farm within the range of their dispersal, as suggested by the CrI not overlapping zero at elevations of 1400 m and above and by the negative trend between corrected elevational shift and elevation (Table 3, Fig. 4). Moreover, juveniles fledged at low elevations showed clear upwards dispersal (as suggested by the CrI not overlapping zero at low elevations) and settled at higher elevations than the average farm within the range of their dispersal (Figs. 3, 4).

**Table 3:** Parameter estimates of the linear mixed models investigating factors affecting dispersal distance and elevational shift of dispersal.

Parameter	Dispersal distance		Corrected elevational shift	
	Estimate	95% CrI	Estimate	95% CrI
**Fixed effects**				
Intercept	7.80	7.28 to 8.31	27.37	− 64.53 to 118.35
Age (adult)	− 1.40	− 2.16 to − 064	− 71.07	− 260.81 to 119.55
Elevation (z-transformed)	− 0.18	− 0.68 to 0.34	− 86.64	− 187.62 to 14.89
Sex (female)	0.03	− 0.42 to 0.49	− 19.30	− 151.27 to 115.07
Elevation x age (adult)	0.16	− 0.34 to 0.66	36.22	− 98.11 to 171.65
Elevation x sex (female)	− 0.02	− 0.47 to 0.43	− 31.04	− 156.28 to 94.86
Age (adult) x sex (female)	0.16	− 0.88 to 1.24	39.29	− 237.59 to 311.12
**Random effects**				
Individual	< 0.01	< 0.01 to < 0.01	0.65	0.48 to 0.87
Location	1.12	0.91 to 1.38	99.64	71.40 to 134.65
Year	0.22	0.14 to 0.33	36.63	21.94 to 55.92

The lower and upper limits of the 95% credible intervals are based on Monte Carlo simulation of the posterior distribution. For the random effects the among-group standard deviation is given. N = 56 dispersal events of 51 individuals from 26 farms.

## Discussion

The long-term mark-recapture study in a small Alpine population of barn swallows revealed clear demographic patterns over a 700 m elevational gradient. First, annual apparent survival of adult breeding birds decreased with increasing elevation towards the upper range limit, in particular in males. Second, breeding dispersal probability of adult females, but not males increased strongly towards the upper range limit. And third, adult and juvenile barn swallows at the highest elevations dispersed to farms situated at elevations lower than expected by random dispersal, while juveniles at low elevations dispersed to farms at elevations higher than expected by random dispersal. By considering more than one demographic parameter at the elevational range limit^4,31^, we show for a highly mobile passerine bird that not only survival is reduced at the upper range limit, but that also breeding dispersal probability is increased and dispersal is directed downwards. Thus, this study provides evidence for a higher turn-over rate of breeding individuals and increased spatial dynamics at the upper range limit.

Unfortunately, we lack reliable long-term data on reproductive output in our Alpine study population of barn swallows. However, in a recent study over 13 Swiss barn swallow populations including our study population we show that though fecundity is increased at high elevations, nestling survival is considerably reduced and start of breeding delayed^29^. A delayed start of breeding is shown to result in a decrease in both the annual number of successful broods and the number of fledglings in successful broods^32^, and thus, in a reduced annual reproductive output^33^. Moreover, since the activity of aerial insects, the main food of barn swallows, strongly depends on temperature^34^, we suggested that spells of cold weather have stronger effects on the reproductive output at high elevations than in lowlands^35,36^. We therefore have good evidence that barn swallows breeding at the upper range limit in the Swiss Alps experience reduced reproductive output and that therefore habitat quality at breeding sites declines at high elevations.

As expected, within-study area dispersal probability was high for juveniles (natal dispersal) and low for adult birds (breeding dispersal), confirming that adult barn swallows are highly faithful to their breeding site^30,37–40^. However, this was only the case at low elevations: within-study area dispersal probability of females strongly increased at elevations approaching the upper range limit. A likely underlying mechanism at least partly responsible for this pattern is the decline in reproductive success at high elevations shown to provoke increased dispersal probabilities of females^30^. In contrast, male dispersal probability within the study area was independent of the elevation of the breeding site. These results suggest that the environmental gradient towards high elevations negatively affecting reproduction results in a spatial gradient of female breeding dispersal and in increased turn-over rates of females compared to males at the upper range limit.

Dispersal at high elevations was directed downwards. Thus, barn swallows preferably selected breeding sites at lower elevations either due to climatic or other environmental gradients changing with elevation. Since in this study all nest sites at both low and high elevations were located in the preferred cowsheds hosting cattle^39,41^, small-scale quality of nest sites can be excluded as a reason for the observed pattern. Settlement decisions towards low elevation might be affected by an increased availability of patches with high density of aerial insects^34,42,43^ or by the prolonged daily and seasonal duration of high insect activity due to temperature gradients^34^. We suppose that these nest site preferences are not only the reason for directed downwards dispersal, but also prevent settlements at farm buildings with cattle at even higher elevations. The preference for breeding sites at lower elevations suggests that immigration of juvenile birds into the study area first occurs at low elevation until a critical breeding density is reached. Later arriving individuals, often individuals of lower quality^39^, then start to select less preferred breeding sites at elevations over 1000 m^44^. This is further supported by the fact that more than 1/3 of the juveniles that fledged at low elevations settled at very high elevation for their first brood. This within-population dispersal and settlement dynamics is in line with ideal despotic settlement processes across the elevational gradient^27,28^.

At low elevations, apparent survival showed the well-known sex- and age-specific patterns of small passerines in continuous habitats. While apparent survival of juveniles (i.e. recruitment) was considerably lower than that of adults also due to reduced first-year survival and higher rates of natal than breeding dispersal^45–47^, males showed higher apparent survival than females^30^. The latter can be explained by higher dispersal rates out of the study area by females than by males after brood loss or reduced reproductive success^30^. However, at high elevations, apparent survival of adult breeding birds declined, and this decline was stronger in males than in females. This pattern can arise due to either increased breeding dispersal out of the study area or reduced true survival at high elevations. The fact that juvenile apparent survival was independent of elevation rejects the hypothesis that low fecundity at high elevations may be associated with increased first-year recruitment rates, as has been suggested for other bird species^48^.

The increased within-study area dispersal rates of females at high elevations suggest that part of the female decline in apparent survival is due to increased dispersal out of the study area. However, the elevation-independent breeding dispersal probability of males does not fit to this explanation, as higher male dispersal rate outside of the study area but not within the study area seems unlikely. This suggests higher mortality at high elevations. A decline in true survival in both sexes could be due to higher reproductive efforts at higher elevations^29^ potentially bearing higher reproductive costs, or because low quality individuals that were outcompeted in the lowlands settle at high elevation. The sex-specific difference then might be due to the fact that males arrive earlier at breeding sites^39,40^ and therefore are more prone to adverse weather conditions in early spring^49–51^.

The demographic gradients in combination with the downwards directed dispersal shown in this study revealed that the population covering an elevational gradient of 700 m shows characteristics of source-sink dynamics resulting in a dispersal-extended upper range limit^18,52^. Similar to source-sink dynamics between distinct populations or patches^31,52,53^, dispersal allows the section of the population at the range limit to persist although it could not persist in the absence of immigration, either from within the population (low elevations) or from different populations. The dispersing and dead breeding birds at high elevations must be replaced to maintain population size at the upper range limit. The results suggest that juveniles from low elevations tend to recruit at high elevations. In addition, since recruitment rates of juveniles remained unchanged and low, only immigration can maintain the number of breeding pairs at high elevations. This is also the case in study areas of continuous barn swallow populations at low elevations^30,32,47^. However, immigration at the upper range limit in this study must be considerably higher than at low elevations.

Increased immigration at high elevations can have several consequences. First, the location of the range limit does not only depend on the environmental gradients, but also on factors affecting the immigration rate to high elevations, i.e. density-dependent effects at low elevations^17,18^. Thus, in years after low reproductive output or annual survival i.e., in years with growth rates λ <  = 1 at low elevations, we expect low numbers of immigrants to high elevations. After several years of such conditions, we predict a descending upper range limit. In contrast, several years of λ > 1 at low elevations might result in a rise of the upper range limit extending the limit even more upwards to elevations with low nest site preference and low reproductive output. This potential shift is supported by the fact that the number of breeding pairs at high elevations declined over the 15-year study period and some of the highest breeding sites remained unoccupied, while the number of breeding pairs at low elevations was stable. The immigrants to high elevations might not only come from low elevations of the same population but also from other populations. Second, the within-population elevational source-sink dynamics is likely to result in spatial structuring of the population by sorting individuals with different traits to different elevations: late arriving immigrants are more likely to end up at high elevations than early arriving immigrants. As immigrants are predominantly first-year breeders^39^, we expect an altered age-structure with higher proportion of first-year breeders at high than low elevations. Moreover, late arriving individuals are often of low quality or in bad body condition^39,49,54^. As first-year breeders and individuals of low quality and body condition show reduced reproductive success and survival^39^, it will be an important next step to investigate whether the accumulation of first-year breeders and individuals of low quality at high elevations will further reinforce the demographic gradients towards the upper range limit. Furthermore, since we have only limited knowledge about the elevational gradient in reproductive success^29^, but reproductive success represents a critical component of the elevational gradients in demography, studies considering all demographic rates are required to understand how dispersal dynamics act together with survival and reproduction. Overall, environmental gradients at mountain slopes in combination with within-population source-sink effects leading to spatial structuring can result in steep gradients of demographic rates.

In conclusion, this study provides evidence that the formation of the upper range limit of barns swallows is based on two mechanisms: preference for low-elevation breeding sites and the immigration to high elevations associated with source-sink effects. We therefore suggest that within-population elevational range shifts of barn swallows and other mobile vertebrates can occur due to factors affecting both habitat selection and immigration to high elevations. The occurrence and speed of the expected shift of the upper range limit depends not only on the improvement of high-elevation habitats due to climate change (i.e. changes affecting environmental gradients^21,55^), but also on the effects of environmental changes (climate and land-use change) on reproduction and survival of the population sections living at low elevations. One of the reasons for the high variation in shift directions and the smaller upwards shifts than expected from regional increase in temperatures in Alpine bird species^20,23,56^ might therefore be the declining population sizes of many bird species at lower elevations.

## Methods

### Study species

The barn swallow is a migrant passerine normally breeding in agricultural landscapes^39^. In the Swiss Alps barn swallows occur frequently from the lowland up to around 1100 m a.s.l., with regular occurrence up to 1300 m a.s.l. However, there are records of broods at exceptionally high elevations, as high as 1900 m a.s.l.^24^. At high elevations, breeding sites are restricted to farm buildings inhabited by cattle because they provide increased food resources and enhanced thermal conditions^29^. Thus, the species’ dependence on specific human structures is particularly high at the upper distribution of the species, and the density of cowsheds is decreasing at high elevations. In our study area, the highest broods were recorded at 1430 m. However, cattle stables and cow sheds in our study area occur also at higher elevations than these broods (up to over 1700 m). Consequently, it is not the availability of stables that determines the upper range limit for this species at our study site. Estimates of natal and breeding dispersal in barn swallows normally are below 10 km and 1 km, respectively^39^.

### Study area and bird ringing

The study was conducted in the Eastern Swiss Alps, in the central part of the Prättigau valley (Canton of Grisons). The research area was c. 10 km in length and 3.5 km in width (i.e., c. 35 km^2^), and included parts of the villages of Schiers, Jenaz, Furna, Luzein, Fideris, Küblis, Conters and Serneus below 1450 m a.s.l. While the surrounding mountains reached elevations of 2000 m a.s.l. and more, the study area covered 63 farms at elevations from the bottom of the valley at 700 m a.s.l. up to 1430 m a.s.l. (mean elevation = 1042 m a.s.l., SD = 224 m). The landscape within the study area was characterized by a mixture of forests, open land, and rural settlements. Most of the fields were used as pastures or hay meadows. Arable land was restricted to the bottom of the valley. The field work took place in the years 1998 to 2012. From 1998 to 2003, we worked at 16 farms in Alpine pastures in a 2 km^2^ study plot in the village of Küblis (at elevations between 1180 and 1330 m a.s.l.). In 2004 the study area was enlarged to the final size. The abundance of farm buildings declined with increasing elevations. Therefore, we mapped all accessible stables within our study sites in order to estimate an expected value of elevational shift under random dispersal to all available nest sites independent of elevation.

During the breeding season, stables and barns of the farms were regularly visited for detecting the barn swallow broods. Juveniles in accessible nests were ringed at the age of 5–15 days. Adults were caught during their rearing periods, usually in the late evening when they rested in or close to the nest, using a hand net, or they were caught with mist nets mounted at the entrances of the buildings. Reproductive output (and ringing of juveniles) was only assessed at the fraction of accessible nests and therefore data on reproductive output were not available in sufficient quality in this study. The study was part of the EURING Swallow Project (https://euring.org/research/swallow-project) investigating factors affecting barn swallow demography at a European scale using a field manual to ensure consistent methods. Bird capturing and ringing, and the Swiss ringing scheme for the EURING Swallow Project was authorised by the Swiss Federal Office for the Environment FOEN with annual licences to JvH and to the volunteer ringers mentioned in the acknowledgements. Capturing and ringing were carried out in accordance with the guidelines and regulations of FOEN. The reporting of this study involving animals follows the recommendations in the ARRIVE guidelines (https://arriveguidelines.org).

### Mark-recapture analysis

To analyse how apparent survival correlates with elevation and age we used a Cormack-Jolly-Seber type of model^57–60^ (n = 1531 ringed individuals (1337 nestlings, 194 adults) and 89 recaptured individuals). In our model, we included linear predictors for apparent survival and for recapture probabilities using the logit-link function. Survival was modelled dependent on age (two classes: first year and older), sex, elevation of breeding (adults) or fledging (juveniles) site and the interactions age x elevation and sex x elevation. We further included a variable that indicated whether the breeding or fledging site was in the centre or at the edge of the study area (location within study area, binomial: edge vs. centre) to account for the fact that individuals being born or breeding at the edge of the study area have a higher chance to leave the study area from one year to the next. In addition, we included year and the farm of origin (breeding or fledging site) as normally distributed random variable in the linear predictor. As predictors for recapture probability, we used age, elevation of the site and their interaction as fixed predictors and year as random effect. Because sex was not known for nestlings without recaptures, we included a categorical model for sex within the mark-recapture model: sex_i_ ~ Categorical(**f**), where **f**_i_ is a vector containing the proportion of males and females, respectively (see Table 4). By adding the information of the unknown sex to the data set we were able to fit the model to data including all individuals while accounting for the unknown sexes. The model estimated the proportion of males among the nestling individuals with unknown sex to be 45%. This value is a reasonable estimate of the nestling sex ratio^62^, and therefore suggests that sex-specific parameters were estimated adequately.

**Table 4:** Description of the models conducted in this study.

Analysis	Response variable	Model structure
Apparent survival	*y*_*it*_: indicator of a capture of individual *i* in year *t*	*y*_*it*_ ~ Bernoulli(*z*_*it*_*p*_*it*_) *z*_*it*_ ~ Bernoulli(*z*_*it*-1_*Φ*_*it*_), with *z*_*it*_ = 1 for *t* = year of first capture for individual *i* logit(*Φ*_*it*_) = *β*_0_[sex_*i*_, age_*it*_] + * β*_1_[sex_*i*_, age_*it*_]*elevation_*it*_ + *β*_2_*edge_*it*_ + *δ*[site_origin_*it*_] + *γ*_*t*_ logit(*p*_*it*_) = *α*_*0*_[age_*it*_] + * α*_*1*_[age_*it*_]*elevation_*it*_ + *ρ*_*t*_ sex_*i*_ ~ Categorical(**f**) *δ*[site_origin] ~ Normal(0, *σ*_*δ*_^2^) *γ*_*t*_ ~ Normal(0,*σ*_*γ*_^2^) *ρ*_*t*_ ~ Normal(0,*σ*_*ρ*_^2^)
Dispersal probability	*y*_*i*_: binary indicator of a dispersal for recapture *i*	*y*_*i*_ ~ Bernoulli(*p*_*i*_) logit(*p*_*i*_) = *β*_0_[sex_*i*_, age_*i*_] + *β*_1_[sex_*i*_]*elevation_*i*_ + *β*_2_*edge_*i*_ + *δ*[site_origin_*i*_] + *γ*[year_*i*_] + *λ*[individual_*i*_] *δ*[site_origin] ~ Normal(0,*σ*_*δ*_^2^) *γ*[year] ~ Normal(0,*σ*_*γ*_^2^) *λ*[individual] ~ Normal(0,*σ*_*λ*_^2^)
Dispersal distances	*y*_*i*_: dispersal distance of dispersal *i*	*y*_*i*_ ~ Normal(*μ*_*i*_) *μ*_*i*_ = *β*_0_[sex_*i*_, age_*i*_] + *β*_1_[sex_*i*_]*elevation_*i*_ + *β*_2_*edge_*i*_ + * δ*[site_origin_*i*_] + *γ*[year_*i*_] + * λ*[individual_*i*_] *δ*[site_origin] ~ Normal(0,*σ*_*δ*_^2^) *γ*[year] ~ Normal(0,*σ*_*γ*_^2^) *λ*[individual] ~ Normal(0,*σ*_*λ*_^2^)
Elevational shift	*y*_*i*_: corrected elevational shift of dispersal *i*	*y*_*i*_ ~ Normal(*μ*_*i*_) *μ*_*i*_ = *β*_0_[sex_*i*_, age_*i*_] + *β*_1_[sex_*i*_]*elevation_*i*_ + *β*_2_*edge_*i*_ + * δ*[site_origin_*i*_] + *γ*[year_*i*_] + * λ*[individual_*i*_] *δ*[site_origin] ~ Normal(0,*σ*_*δ*_^2^) *γ*[year] ~ Normal(0,*σ*_*γ*_^2^) *λ*[individual] ~ Normal(0,*σ*_*λ*_^2^)

### Dispersal analysis

Analysis of dispersal probability was restricted to birds with at least one recapture (n = 126 recapture events of 89 individuals). A dispersal event was defined as breeding attempt at a different farm than the last breeding or fledging event. A farm was a clearly separated entity of stables and sheds close together and distance between farms was normally > 300 m. We analysed this binary indicator variable for dispersal (recaptured at the initial farm = 0, recaptured at a new farm = 1) with a logistic regression (binomial error distribution and logit-link function was assumed, Table 4). As predictor variables we used age, sex, elevation of the farm before the dispersal event, location within the study area (edge vs. centre) and the two-way interactions of elevation with age and sex. Each recapture occasion represented a data point. Thus, individuals with more than one recapture were measured repeatedly. Additionally, dispersal characteristics could be influenced by characteristics of the farm or the year. We therefore included individual, farm of origin, and year as random factors in linear mixed models to account for these correlations.

Analyses of dispersal distances and elevational shifts within the study area were restricted to birds with at least one dispersal event within the study area (n = 56 dispersal events of 51 individuals). Elevational shift could be biased by the fact that birds of high elevations had higher probability to descend because they had less opportunity to climb (and vice versa). Consequently, birds at high elevations may have dispersed to lower elevations also when they dispersed randomly to the available farms. Therefore, we calculated the mean elevation of the available farms within the radius of the dispersal distance of the respective bird. Thus, a bird dispersed to a farm at higher or lower elevation than the average farm within the range of dispersal. This elevational difference was denoted corrected elevational shift.

For analysing effects on dispersal distances and on corrected elevational shifts the normal distribution was assumed and the same predictors as for the analyses of dispersal probability were included in the linear mixed effects models (except “location within the study area” which was excluded, Table 4). The models were fitted to the data using the functions lmer and glmer from the package lme4^61^ in the program R^63^. Before fitting the models, all numeric predictors were centred and scaled to a variance of one. We quantified uncertainty of the parameter estimates using Bayesian methods as implemented in the function sim from the package "arm"^64^. Thereby, flat prior distributions were assumed for each model parameter, and the posterior distributions were described using Monte Carlo simulation. The 2.5% and 97.5% quantiles of 2000 simulated values were used as the limits of the 95% credible intervals (CrI).

## Data Availability

The datasets generated and analysed are publicly available at the *vogelwarte.ch Open Repository and Archive* (10.5281/zenodo.5336309).
